# Supplementary material for: Solvent-free processing of lignin into robust room temperature phosphorescent materials
Source: Nat Commun. 2025 Mar 12;16:2455. doi: 10.1038/s41467-025-57712-x (PMC11904197; doi:10.1038/s41467-025-57712-x)
Supplement: Supplementary file 1 — Supplementary Information [file 41467_2025_57712_MOESM1_ESM.pdf]

Supplementary information

Solvent-free processing of lignin into robust room temperature phosphorescent materials

Min Wang[1], Wei-Ming Yin[1], Yingxiang Zhai[1], Jingyi Zhou[1], Shouxin Liu[1], Jian Li[1], Shujun Li*[1], Tony D. James*[2,3], Zhijun Chen*[1]

1. Key Laboratory of Bio-based Material Science & Technology, Northeast Forestry University, Ministry of Education, Harbin 150040, China.
2. Department of Chemistry, University of Bath, BA2 7AY Bath, UK.
3. School of Chemistry and Chemical Engineering, Henan Normal University, Xinxiang 453007, P. R. China.

E-mail: T.D.James@bath.ac.uk; lishujun@nefu.edu.cn; chenzhijun@nefu.edu.cn.

Supplementary methods

Chemicals and Materials

Enzymatic hydrolysis lignin (EL) was purchased from Longlive Biological Technology Co.Ltd. (Shandong, China), it was purified and extracted with ethanol before use. Alkali lignin (AL) and sodium lignosulfonate (SL) were purchased from Aladdin (Shanghai, China). The monomer 2-Hydroxyethyl acrylate (HEA, 96%) and Urethane dimethacrylate, mixture of isomers (UDMA, ≥ 97%) were purchased from Aladdin. The monomer HEA was passed through a neutral alumina column to remove the inhibitor before using. Organic reagents, including ethanol (99.5%), methanol (99.5%), dichloromethane (99.5%), acetonitrile (99.5%), tetrahydrofuran (99.5%) and ethyl acetate (99.5%), were purchased from Tianjin Tianli Chemical Reagent Co., Ltd. Rhodamine B (RhB, > 98%) was obtained from Aladdin. Radical scavenger 5,5-dimethyl-1-pyrroline N-oxide (DMPO, 97%) was purchased from Aladdin (Shanghai, China). Ethylene glycol (99%) and ethyl acrylate (99%) were purchased from Shanghai Adamas Reagent Co., Ltd. 2,2'-Azobis(2-methylpropionitrile) (AIBN, 99%) was purchased from Aladdin. LED-UV curing lamp (photocuring wavelength =365 nm, optical density =170 mW/cm^2) was purchased from Zhongshan UV curing lighting appliance factory in Taobao (Alibaba, Hangzhou, China). Trimethylolpropane triacrylate (TMPTA, ≥ 85%), Triethylene glycol dimethacrylate (TEGDA, ≥ 95%), Tripropylene glycol diacrylate (TPGDA, ≥ 90%), N-Hydroxy-5-norbornene-2,3-dicarboximide (e-HNDI, 99%) and Chromium (lll) acetylacetonate ($Cr(acac)_3$, 97%) were purchased from Aladdin (Shanghai, China). Deuterated dimethyl sulfoxide (DMSO-d6, D, 99.8%), Deuterated chloroform ($CDCl_3$, D, 99.8%), Deuteropyridine (Pyridine-D5, D, 99.5%) and 2-chloro-4,4,5,5-tetramethyl-1,3,2-dioxaphospholane (Cl-TMDP, 95%) were purchased from Adamas-beta.

Characterizations

Fluorescent spectra, RTP emission spectra and lifetime decay curves were recorded by a FLS1000 photoluminescence spectrometer (Edinburgh Instruments, Livingston, UK) equipped with a xenon lamp and a one-microsecond lamp (detector: photoelectric multiplier, 200 nm < λ < 1700 nm). Afterglow emission spectra were recorded after a 10 ms delay. The temperature was controlled using an OX135QX cryostat (Oxford Instruments plc, Abingdon, UK). Electron spin resonance (ESR, Bruker A300, Germany) was used to detect reactive free radicals. Test setting was a central field of 3510 G and a scanning width of 300 G. The active species capture agent was DMPO.

The hydroxyl radicals (·OH) in the prepolymer mixture were detected before and after illumination, respectively. The tensile strength of polymerized membrane was measured by a UTM2203 universal testing machine (Shenzhen SUNS Technology Stock Co. Ltd., Shenzhen, China), with a 100 N load cell. The thermal stability of polymerized films was measured by thermogravimetric analysis instrument (TGA Q50, TA USA) under an argon atmosphere, test temperature from room temperature to 600 °C and heating rate was set at 10 $°C·min^{-1}$. The thermodynamic stability of dynamic polymerized films was measured by thermomechanical analyzer (DMA Q800, TA USA) under a nitrogen atmosphere with stretch mode, test temperature was set from room temperature to 260 °C and heating rate was 10 $°C·min^{-1}$. Test frequency was 1 Hz. Fourier transform infrared (FT-IR) spectra were recorded using a Nicolette 6700 S3 FT-IR spectrometer (ThermoFisher Scientific, USA) from 400 cm^{-1} to 4000 cm^{-1}, using the attenuated total reflection (ATR) method. The double bond conversion (DBC) was calculated by measuring the peak area changing with the peaks of C=C stretching vibration at 1637 cm^{-1}. All photos and videos were taken using a mobile phone. GPC was recorded by a gel permeation chromatograph (Agilent Pl-gpc-50) with DMSO as the mobile phase, and PMMA as the standard substance. ^{31}P NMR was measured using a nuclear magnetic resonance spectrometer (AVANCEIIIHD 500MHz). 20 mg of the sample was dissolved in the mixture of Pyridine-D_5 and $CDCl_3$ (1.6:1, v/v). Then 100 μL internal standard of e-HNDI solution (22.7 mg/mL in Pyridine-D5/$CDCl_3$ (1.6:1, v/v)), 50 μL relaxation reagent of $Cr(acac)_3$ (11.8 mg/mL in Pyridine-D5/$CDCl_3$ (1.6:1, v/v)) and 100 μL of phosphorylation reagent (Cl-TMDP) were added. As long as a homogeneous solution was formed, the solution was analyzed by ^{31}P-NMR immediately[1]. The hardness and Young's modulus was tested using a Nanoindentor (Bruker HYSITRON TI 980). The contact angle and surface tension were determined by a contact angle/surface tension measuring instrument (Dataphysics OCA20). The polymerized products used for ^{31} P NMR and GPC were washed by THF to remove the unreacted HEA monomer.

Simulation methods

The geometries of molecule lignin, HEA, UDMA, polymer and complexes of lignin + HEA, lignin + UDMA and lignin + Polymer were all optimized under the framework of density of functional theory with B3LYP[2-4] functional and 6-31G(d,p)[5,6] basis set. In order to describe the dispersion interaction, the DFT-D3 dispersion correction method was also used in the calculation. The symmetry-adapted perturbation theory (SAPT)[7]

method implemented in Psi4 program[8,9] which is one of the most popular and state-of-art energy decomposition analysis (EDA) methods was adopted to investigate the nature of noncovalent interactions between HEA or UDMA and lignin. Considering that this system was too large to perform expensive high-order perturbation calculations, the scaled SAPT0 method (sSAPT0)[10,11] and jun-cc-pVDZ basis set was used to perform the energy decomposition analysis.

In order to analyze the intermolecular interactions, the Independent gradient model (IGM)[12] analysis was performed using the Multiwfn program[13] to study the weak interaction visually. The result was also rendered using the VMD program[14].

Supplementary Figures

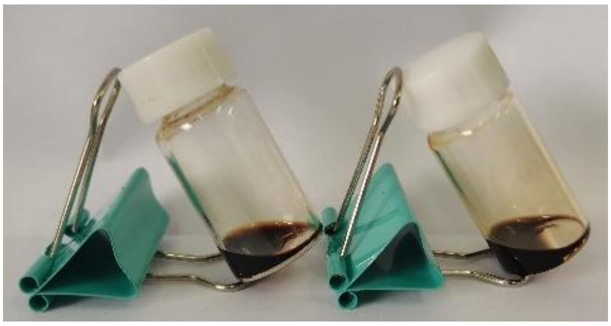

Supplementary Fig. S1 Photographs of homogeneous solution of lignin dissolved in HEA at concentrations of 10 wt% (left) and 15 wt% (right).

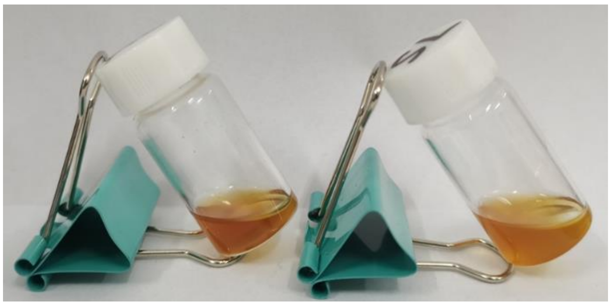

Supplementary Fig. S2 Photographs of AL (left) and SL (right) dissolved in HEA (1 wt%).

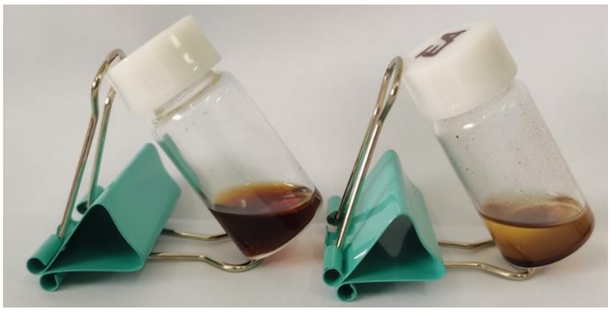

Supplementary Fig. S3 Photographs of lignin dissolved in ethylene glycol (left) and ethyl acrylate (right) (1 wt%).

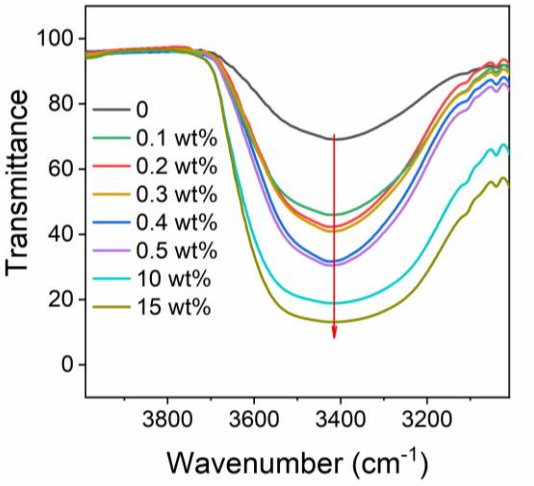

Supplementary Fig. S4 FT-IR spectra for different content of lignin in HEA.

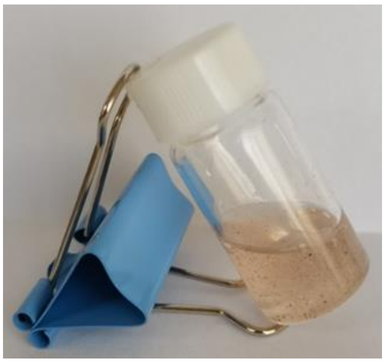

Supplementary Fig. S5 Photograph of lignin dispersed in UDMA (1 wt%).

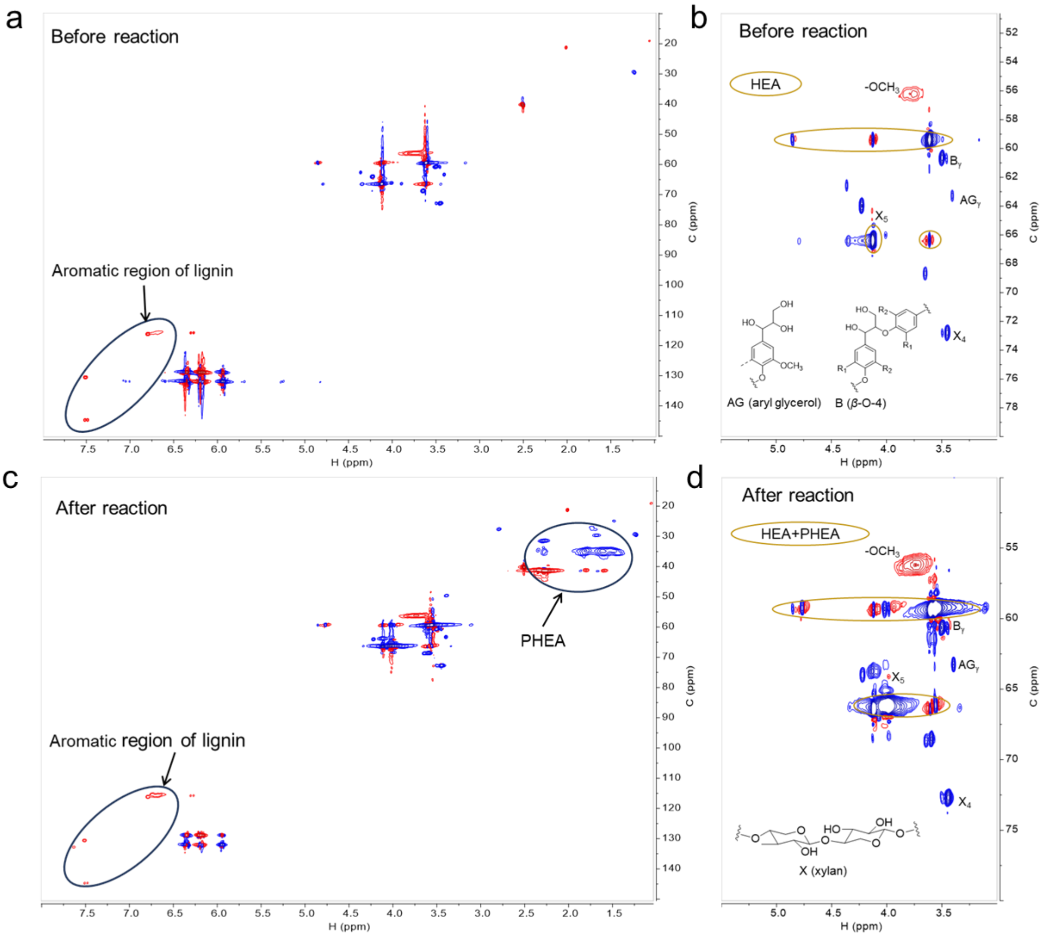

Supplementary Fig. S6 2D HSQC of the mixtures consisting of lignin (15 mg) and HEA (85 mg) in DMSO-d_6 (0.7 mL) before (b) and after (d) UV irradiation for 6 h.

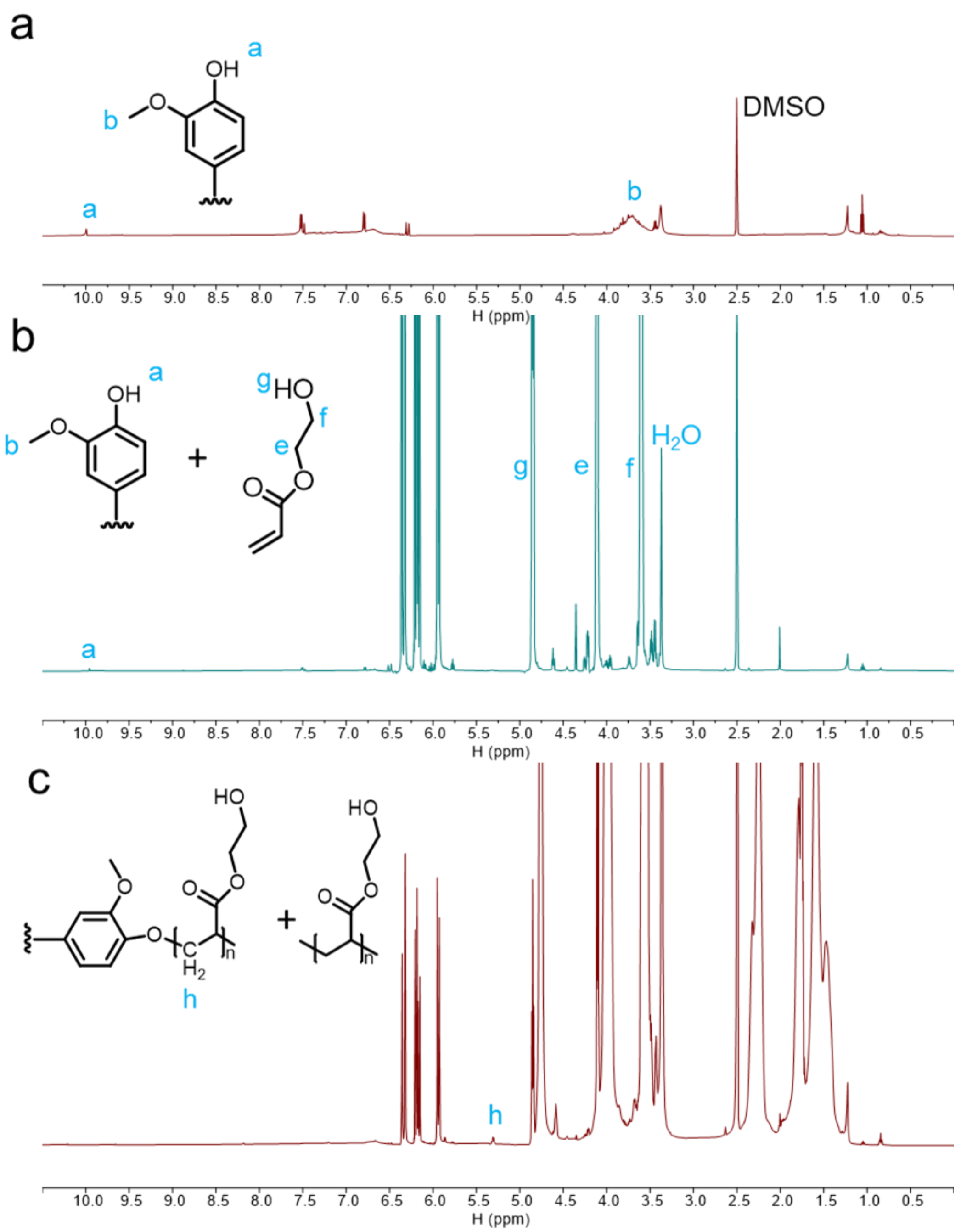

Supplementary Fig. S7 ^{1}H NMR of lignin (a) and the mixtures consisting of lignin (7.5 mg) and HEA (42.5 mg) in DMSO-d_6 (0.7 mL) before (b) and after (c) UV irradiation for 6 h.

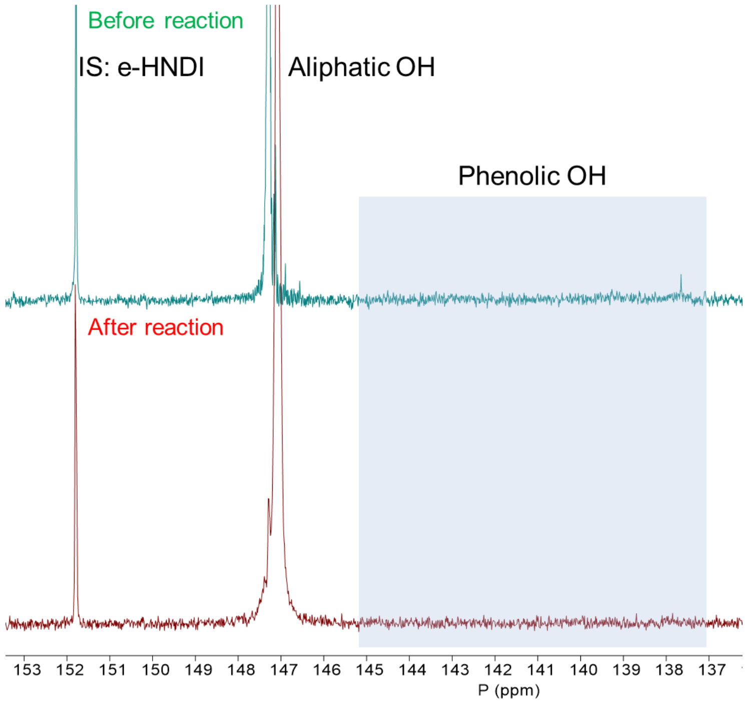

Supplementary Fig. S8 ^{31}P NMR spectra of the mixtures consisting of lignin (7.5 mg) and HEA (42.5 mg) in Pyridine-D_5/$CDCl_3$ (1.6:1, v/v) before and after UV irradiation for 6 h.

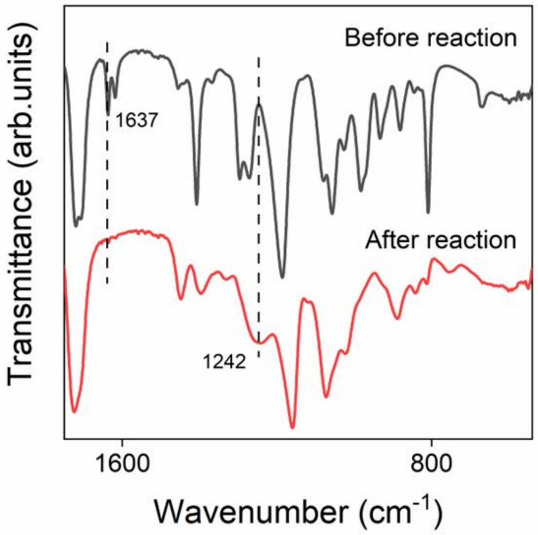

Supplementary Fig. S9 FT-IR spectra of the mixtures consisting of lignin (7.5 mg) and HEA (42.5 mg) before and after UV irradiation for 6 h.

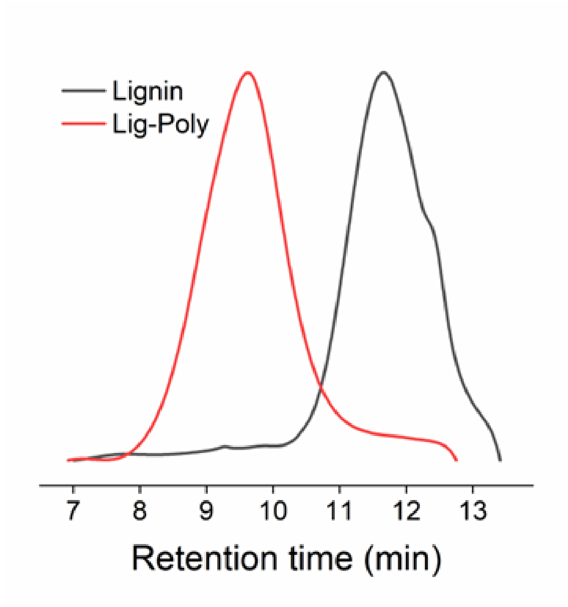

Supplementary Fig. S10 GPC trace of the raw lignin and the mixtures consisting of lignin (7.5 mg) and HEA (42.5 mg) after UV irradiation for 6 h.

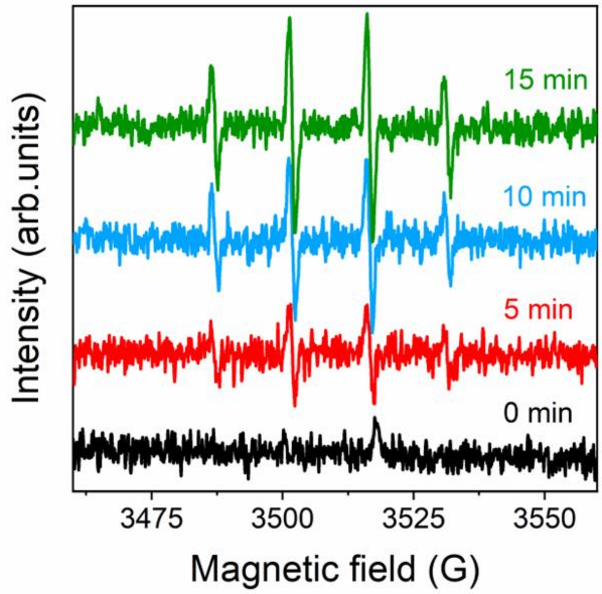

Supplementary Fig. S11 ESR spectra of lignin in a mixture of HEA and UDMA upon UV irradiation for 0 min (black line), 5 min (red line), 10 min (blue line) and 15 min (green line).

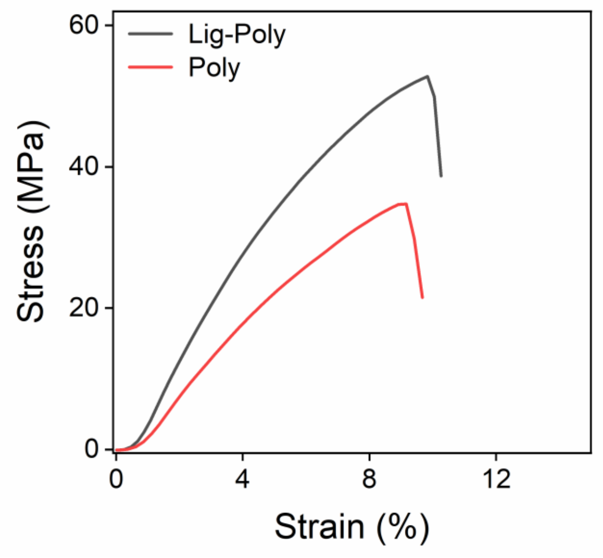

Supplementary Fig. S12 Mechanical properties of **Lig-Poly** (black line) and **Poly** (red line).

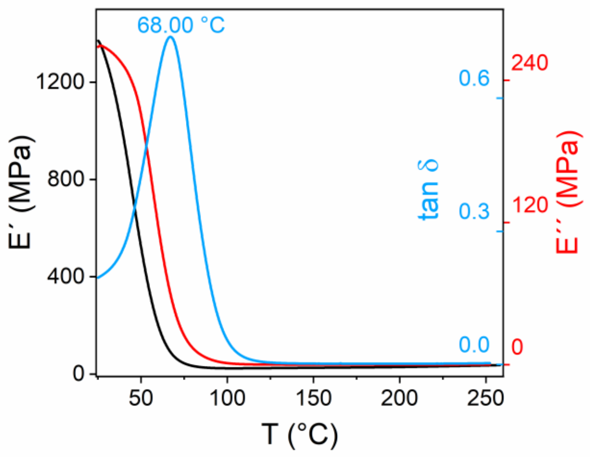

Supplementary Fig. S13 DMA of Lig-Poly.

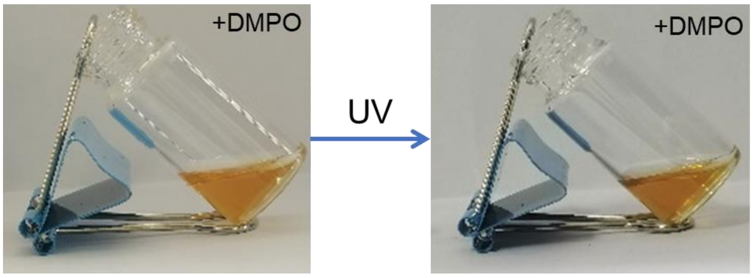

Supplementary Fig. S14 Photographs of precursors with DMPO before (left) and after (right) irradiation of UV light.

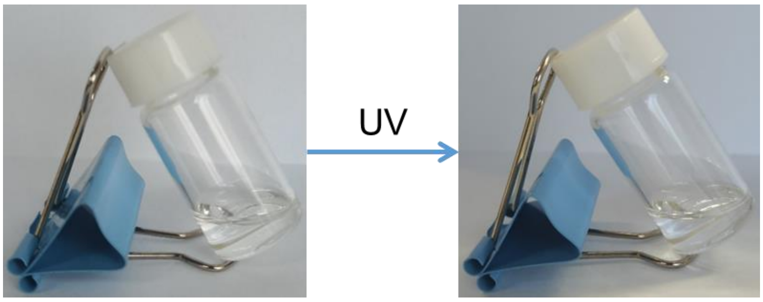

Supplementary Fig. S15 Photographs of precursors without lignin before (left) and after (right) irradiation.

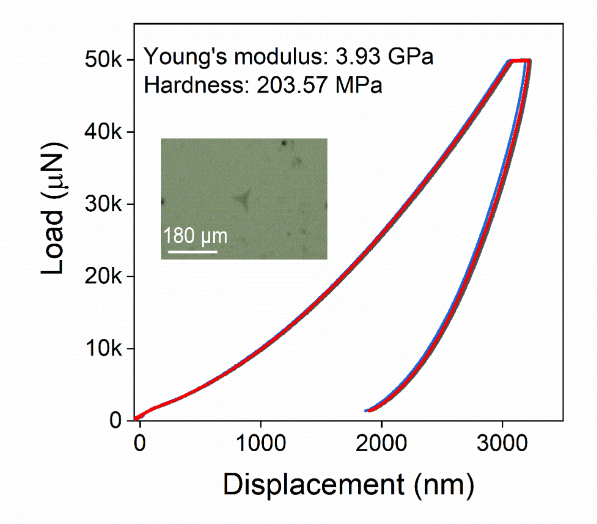

Supplementary Fig. S16 Mechanical properties of Lig-Poly.

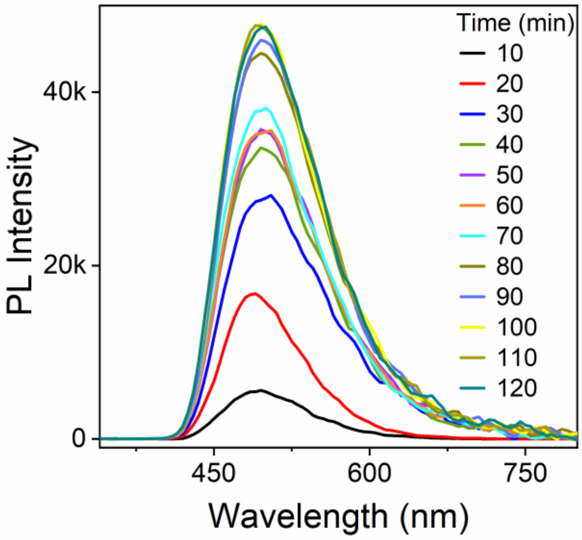

Supplementary Fig. S17 RTP emission spectra of Lig-Poly under different exposure times excited by 320 nm light.

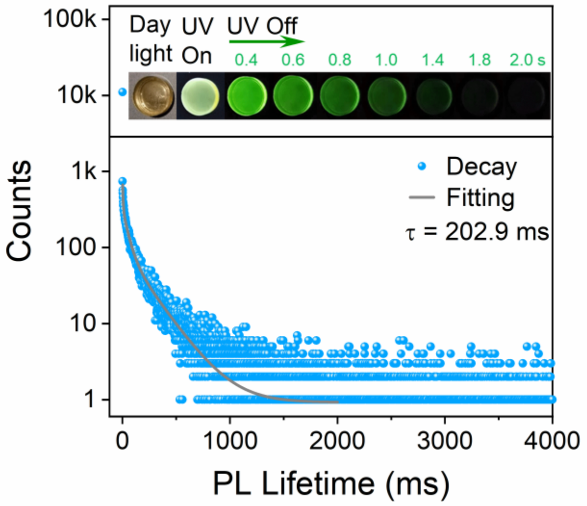

Supplementary Fig. S18 RTP decay curve of Lig-Poly, inset: images of Lig-Poly in daylight, under UV excitation and after turning off the UV light for two seconds.

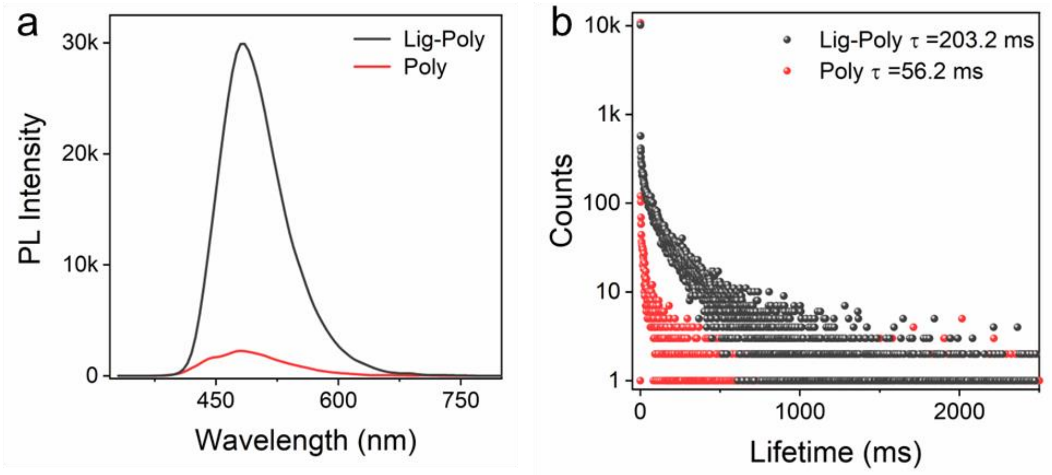

Supplementary Fig. S19 a) Phosphorescence emission spectra of Lig-Poly (black line) and **Poly** (red line). b) Lifetimes of Lig-Poly (black point) and Poly (red point) (λex.= 320 nm, λcollected = 500 nm).

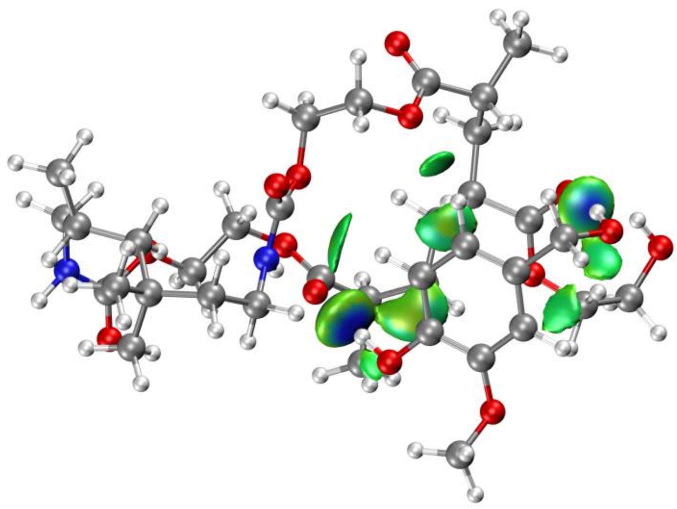

Supplementary Fig. S20 Calculated interaction model of lignin in Lig-Poly.

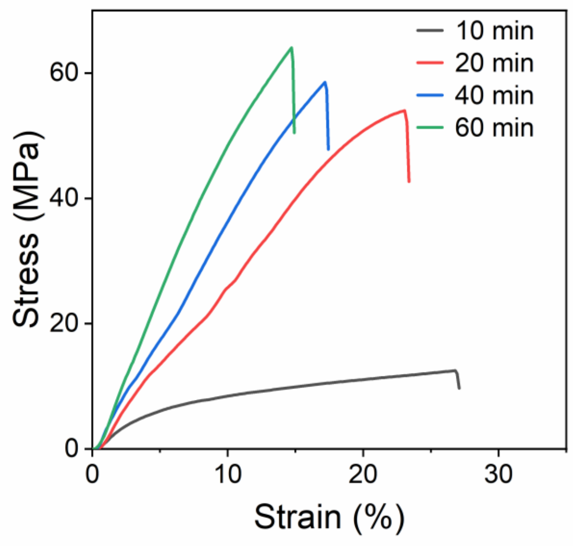

Supplementary Fig. S21 Mechanical properties of Lig-Poly upon UV irradiation for 10 min (black line), 20 min (red line), 40 min (blue line) and 60 min (green line).

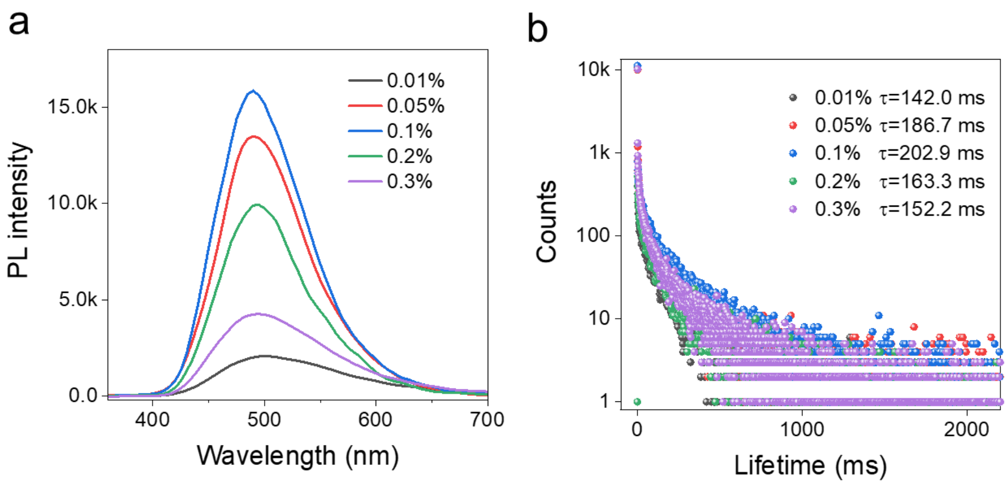

Supplementary Fig. S22 Emissions (a) and RTP lifetimes (b) of Lig-Poly made using different amounts of lignin. In all cases λexc.= 320 nm, λcollected= 500 nm, delay time = 10 ms.

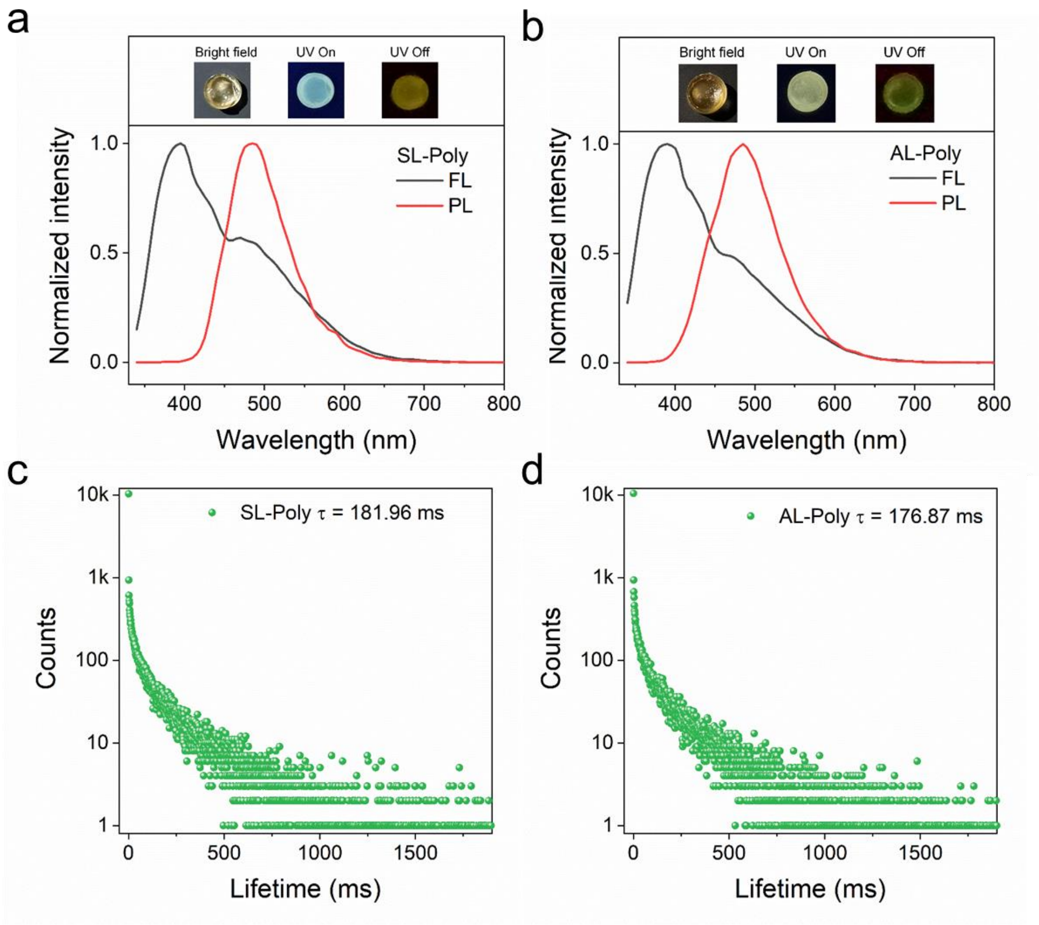

Supplementary Fig. S23 RTP emission spectra and lifetimes of Lig-Poly prepared by different kinds of lignin. Standard (black line) and delayed (red line) emission spectra of a) SL-Poly (sodium lignosulfonate) and b) AL-Poly (alkaline lignin), Inset: the images of polymers in daylight (left), upon excitation by UV light source (middle) and after switching off the UV light source (right); Phosphorescence lifetime of c) SL-Poly and d) AL-Poly. In all cases λexc. = 320 nm, λcollected = 500 nm, delay time = 10 ms.

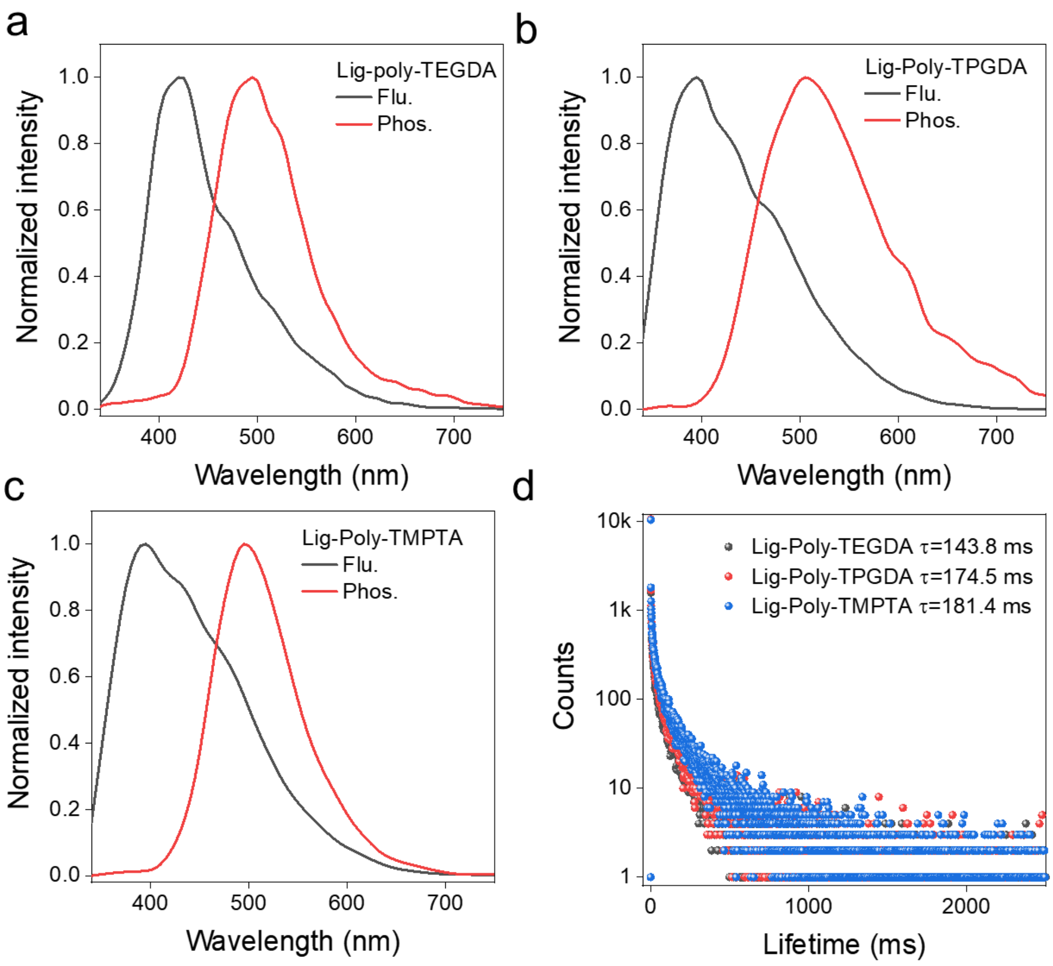

Supplementary Fig. S24 Fluorescence and phosphorescence emission spectra and RTP lifetimes of materials made by other crosslinkers. Fluorescence (black line) and phosphorescence (red line) emission spectra of a) Lig-Poly-TEGDA made with crosslinker TEGDA; b) Lig-Poly-TPGDA made with crosslinker TPGDA; c) Lig-Poly-TMPTA made with crosslinker TMPTA; d) RTP lifetimes of materials made by these three crosslinkers. In all cases λexc.= 320 nm, λcollected= 500 nm, delay time = 10 ms.

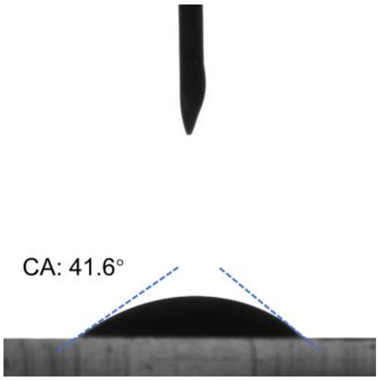

Supplementary Fig. S25 The water contact angle of the cured Lig-Poly.

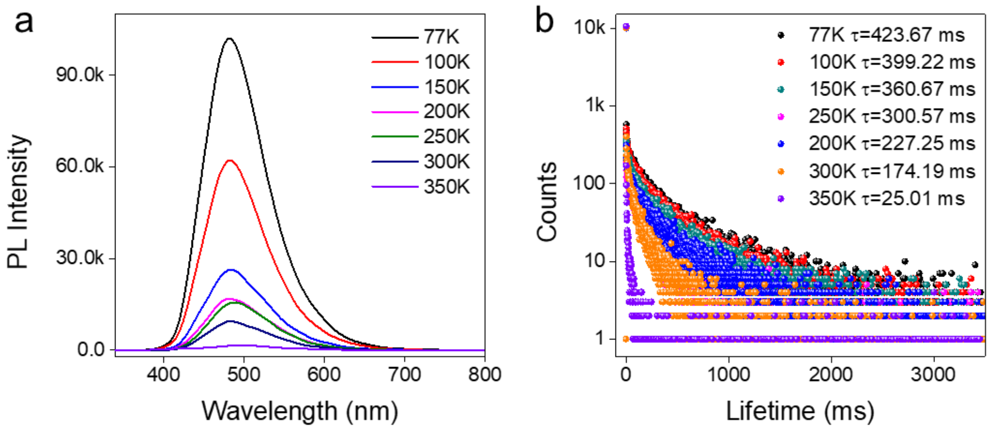

Supplementary Fig. S26 Temperature dependent a) Phosphorescence spectra and b) Lifetime of Lig-Poly (λex.= 320 nm, λcollected = 500 nm).

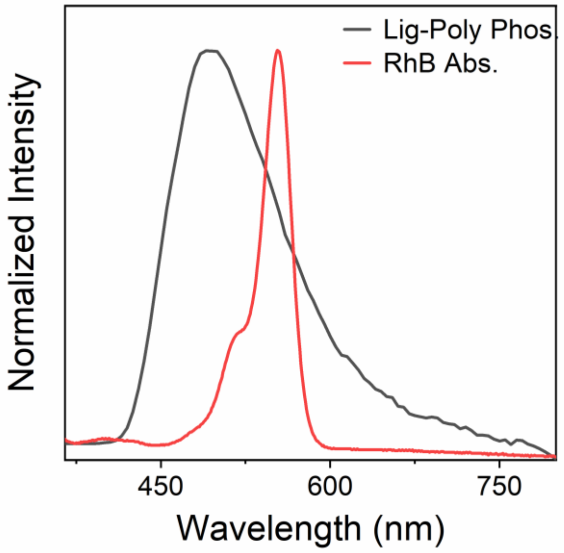

Supplementary Fig. S27 Phosphorescence emission of Lig-Poly (black line) and absorbance of RhB (red line).

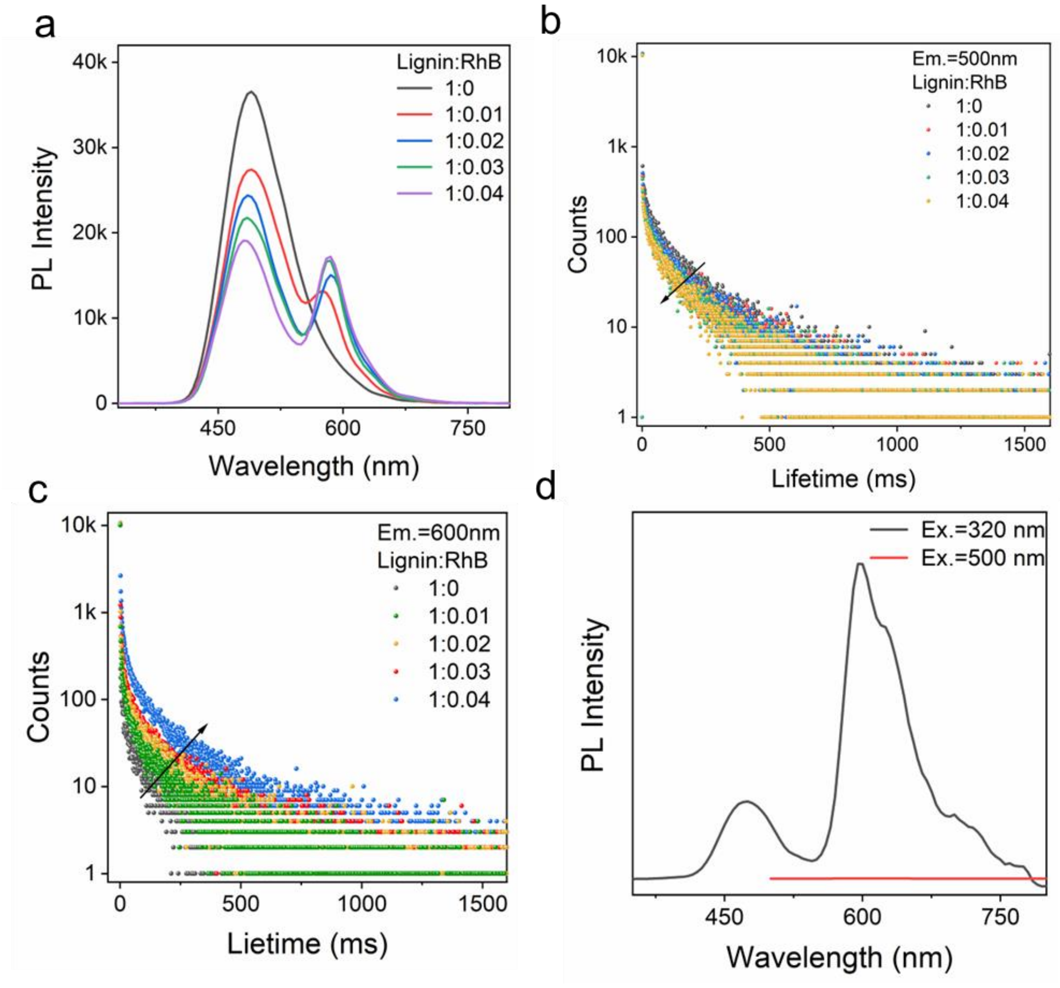

Supplementary Fig. S28 RTP emission and lifetime of Lig-Poly/RhB with different RhB loading contents. a) Phosphorescence emission spectra of Lig-Poly/RhB (λexc.= 320 nm); b) Lifetime decay plots of Lig-Poly/RhB (λcollected= 500 nm) and c) Lifetime decay plots of Lig-Poly/RhB (λcollected= 600 nm) with increasing the doping concentration of RhB; d) Phosphorescence emission spectra of Lig-Poly/RhB upon direct excitation (λexc.= 500 nm, red line) and upon indirect excitation (λexc.= 320 nm, black line), the delay time was 10 ms.

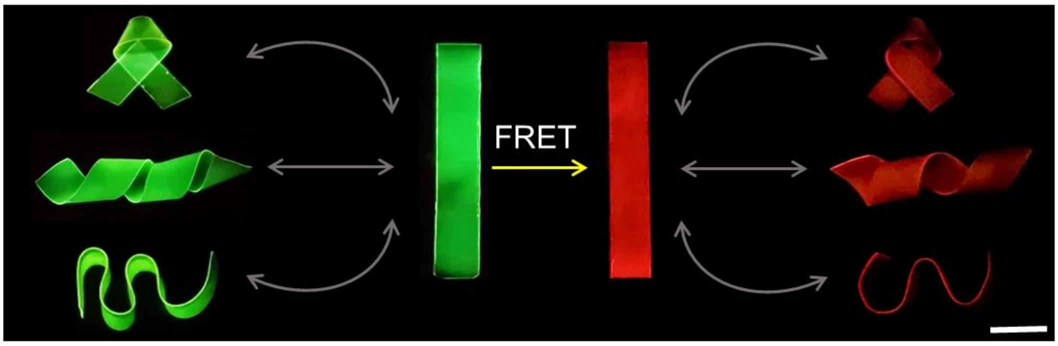

Supplementary Fig. S29 Phosphorescent emission images of Lig-Poly and Lig-Poly/RhB with different shapes formed under photothermal treatment (scale bar = 1 cm).

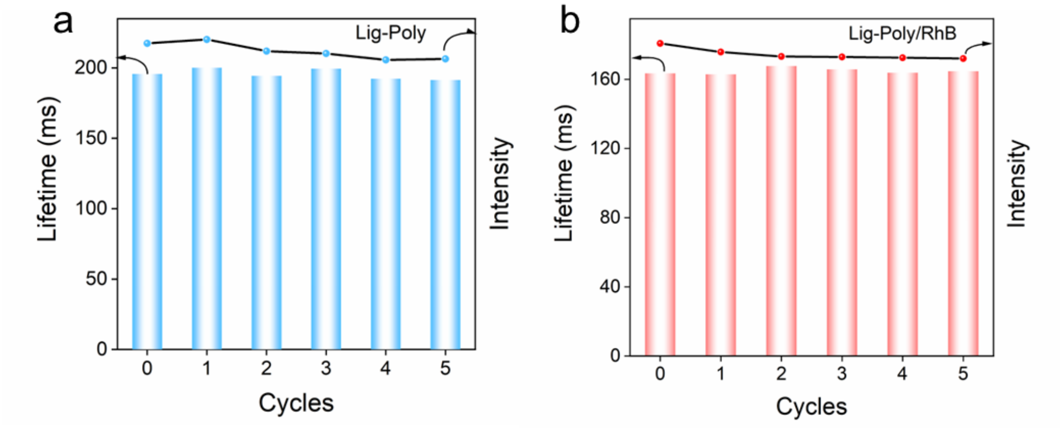

Supplementary Fig. S30 Phosphorescence intensity and lifetime after 5 cycles of thermal treatment. a) RTP emission spectra (black line) and lifetime (blue column) of Lig-Poly excited by 320 nm light. b) RTP emission spectra (black line) and lifetime (red column) of Lig-Poly/RhB excited by 320 nm light.

Supplementary Tables

Supplementary Table S1 Simulation calculation results of intermolecular interaction between EL and HEA, EL and UDMA, EL and HEA with UDMA, EL and polymer respectively.

	Electrostatics [kJ/mol]	Exchange [kJ/mol]	Induction [kJ/mol]	Dispersion [kJ/mol]	Total kJ/mol]
EL-HEA	-83.06	75.83	-23.94	-29.45	-60.62
EL-UDMA	-65.36	78.48	-19.25	-43.18	-49.31
EL-HEA+UDMA	/	/	/	/	-92.64
EL-Polymer	-101.99	124.35	-29.88	-87.77	-95.30

Supplementary Table S2 Summary of energy transfer (Φ_{et}) efficiency.

Acceptor	Donor (Lignin) and acceptor (RhB) Ratio (w:w)	Average lifetime (ms) of Lig-Poly/RhB at 500 nm (λ_{ex} = 320 nm)	Energy Transfer Efficiency (%)
/	1:0	203.2	0
RhB	1:0.01	172.0	15.4
RhB	1:0.02	165.9	18.4
RhB	1:0.03	158.5	22.0
RhB	1:0.04	141.5	30.4

Supplementary References

1 Meng, X. *et al.* Determination of hydroxyl groups in biorefinery resources via quantitative ^{31}P NMR spectroscopy. *Nat. Protoc.* **14**, 2627-2647 (2019).

2 Becke, A. D. Density-functional exchange-energy approximation with correct asymptotic behavior. *Phys. Rev. A* **38**, 3098-3100 (1988).

3 Lee, C., Yang, W. & Parr, R. G. Development of the Colle-Salvetti correlation-energy formula into a functional of the electron density. *Phys. Rev. B* **37**, 785-789 (1988).

4 Becke, A. D. Density-functional thermochemistry. I. The effect of the exchange-only gradient correction. *J. Chem. Phys.* **96**, 2155-2160 (1992).

5 Petersson, G. A. *et al.* A complete basis set model chemistry. I. The total energies of closed-shell atoms and hydrides of the first-row elements. *J. Chem. Phys.* **89**, 2193-2218 (1988).

6 Petersson, G. A. & Al-Laham, M. A. A complete basis set model chemistry. II. Open-shell systems and the total energies of the first-row atoms. *J. Chem. Phys.* **94**, 6081-6090 (1991).
7 Szalewicz, K. Symmetry-adapted perturbation theory of intermolecular forces. *WIRES Comput. Mol. Sci.* **2**, 254-272 (2012).
8 Jeziorski, B., Moszynski, R. & Szalewicz, K. Perturbation Theory Approach to Intermolecular Potential Energy Surfaces of van der Waals Complexes. *Chem. Rev.* **94**, 1887-1930 (1994).
9 Parker, T. M., Burns, L. A., Parrish, R. M., Ryno, A. G. & Sherrill, C. D. Levels of symmetry adapted perturbation theory (SAPT). I. Efficiency and performance for interaction energies. *J. Chem. Phys.* **140** (2014).
10 Hohenstein, E. G., Parrish, R. M., Sherrill, C. D., Turney, J. M. & Schaefer, H. F., III. Large-scale symmetry-adapted perturbation theory computations via density fitting and Laplace transformation techniques: Investigating the fundamental forces of DNA-intercalator interactions. *J. Chem. Phys.* **135** (2011).
11 Hohenstein, E. G. & Sherrill, C. D. Density fitting and Cholesky decomposition approximations in symmetry-adapted perturbation theory: Implementation and application to probe the nature of π-π interactions in linear acenes. *J. Chem. Phys.* **132** (2010).
12 Lefebvre, C. *et al.* The Independent Gradient Model: A New Approach for Probing Strong and Weak Interactions in Molecules from Wave Function Calculations. *ChemPhysChem* **19**, 724-735 (2018).
13 Lu, T. & Chen, F. Multiwfn: A multifunctional wavefunction analyzer. *J. Comput. Chem.* **33**, 580-592 (2012).
14 Humphrey, W., Dalke, A. & Schulten, K. VMD: visual molecular dynamics. *J. Mol. Graph. Model.* **14**, 33-38 (1996).
